# A Wild Rice Rhizobacterium *Burkholderia cepacia* BRDJ Enhances Nitrogen Use Efficiency in Rice

**DOI:** 10.3390/ijms231810769

**Published:** 2022-09-15

**Authors:** Zheng Li, Ahmed R. Henawy, Asmaa A. Halema, Qiuling Fan, Deqiang Duanmu, Renliang Huang

**Affiliations:** 1 State Key Laboratory of Agricultural Microbiology, College of Life Science and Technology, Huazhong Agricultural University, Wuhan 430070, China; 2 National Engineering Research Center of Rice (Nanchang), Key Laboratory of Rice Physiology and Genetics of Jiangxi Province, Rice Research Institute, Jiangxi Academy of Agriculture Sciences, Nanchang 330200, China; 3 Department of Microbiology, Faculty of Agriculture, Cairo University, Giza 12613, Egypt; 4 Department of Genetics, Faculty of Agriculture, Cairo University, Giza 12613, Egypt

**Keywords:** plant growth-promoting rhizobacterium, biological nitrogen fixation, nitrogen use efficiency, wild rice, *Burkholderia*, comparative genomics

## Abstract

Rice domestication has dramatically improved its agronomic traits, albeit with unavoidable significantly reduced genetic diversity. Dongxiang common wild rice, the wild rice species distributed in northernmost China, exhibits excellent resistance against stress and diseases and provides a rich genetic resource for rice breeding. Most of the studies focus on the function of the plant genes, often disregarding the role of the root microbes associated with the plants. In this work, we isolated a *Burkholderia* strain from the root of Dongxiang wild rice, which we identified as *Burkholderia cepacia* BRDJ, based on a phylogenetic analysis. This strain promoted the rice growth under greenhouse conditions. The grain yield was higher in a rice line containing a small genomic fragment derived from the Dongxiang wild rice, compared to the *indica* rice cultivar Zhongzao 35. This new strain also increased the plant biomass under limiting nitrogen conditions. Interestingly, this strain had a differential effect on *indica* and *japonica* rice varieties under full nitrogen supply conditions. By genome sequencing and comparison with another two *B. cepacia* strains, we observed enriched genes related with nitrogen fixation and phytohormone and volatiles biosynthesis that may account for the growth-promoting effects of the BRDJ. BRDJ has the potential to be used as a biofertilizer in promoting nitrogen use efficiency and overall growth in rice.

## 1. Introduction

Dongxiang wild rice (*Oryza rufipogon* Griff., hereafter referred to as DXWR) is the northernmost (116°36′ E, 28°14′ N) extant ancestor of cultivated rice (*Oryza sativa* L.) ever found in the world [1] and can survive at temperatures as low as –12.8 °C [2]. The Rice Research Institute of the Jiangxi Academy of Agricultural Sciences established an in situ reserve in Dongxiang County in 1980 [3] and has sampled and preserved 252 plants from the nine original DXWR habitats and established an ex situ nursery in Nanchang [4]. DXWR harbors valuable traits conferred by its rich genetic diversity. As the northernmost distributed common wild rice in the world, this wild rice contains superior genes that cultivated rice varieties either do not possess, or have lost [5], which are involved in cold tolerance [2], drought resistance [6], disease resistance, and symbiosis [7]. Discovering and introducing these superior genes into rice cultivars is of great practical importance to improve rice yield, resistance, and quality. While the associated rhizosphere and phyllosphere microorganisms coexisting with wild rice may contribute to these superior traits [8], relevant studies are lacking in exploring this connection in detail.

The rhizosphere is an important site for the exchange of materials between plants and their external environment. The growth and activities of plants influence the chemical, physical, and biological characteristics of the soil around their root systems [9,10]. The plant rhizosphere microorganisms and endophytes occupy certain ecological niches in the root–soil–microbe interaction, and these microorganisms can compete for nutrients with pathogenic bacteria in an antagonistic relationship, so that the pathogens are deprived of nutrients and die [11]. Plant growth–promoting rhizobacteria (PGPRs) promote plant growth, increase crop yield, and protect plants against diseases using various mechanisms. They can live in the plant rhizosphere (outside roots, in the surrounding soil), rhizoplane (the root surface), and/or endorhizosphere (inside roots). Considering these benefits, the PGPRs show great promise as a biofertilizer [12].

As beneficial bacteria, PGPRs colonize the roots of many plants such as wheat (*Triticum aestivum*) [13], maize (*Zea mays*) [14], rice [15], and tomato (*Solanum lycopersicum*) [16]. In the soil, these bacteria synthesize growth-promoting substances, such as gibberellins and cytokinins, to alter the root architecture and promote plant development [17,18], as well as increasing the plant’s systemic resistance against pathogens [19].

*Burkholderia* occupy surprisingly diverse ecological niches such as the soil, water bodies, and plant roots. Walter Hagemeyer Burkholder, an American plant pathologist, first discovered the strain in 1949 as the causative agent of onion (*Allium cepa*) stem rot and named the strain *Pseudomonas onionis* [20]. In 1992, Yabuuchi and colleagues formally grouped the bacterium and six other *Pseudomonas* belonging to the same rRNA group into a new genus called *Burkholderia* spp. [21]. The genus comprises 127 published species names [22] and can be divided into at least three major clades [23]. One clade includes the *Burkholderia cepacia* complex (Bcc), the *B. pseudomallei* group, *B. gladioli*, *B. plantarii*, and *B. glumae*, while the second clade includes *B. glathei* and closely related species. The third clade includes the *B. xenovorans* group, which includes many beneficial microorganisms [24].

*B. cepacia* is of great research importance in the field of medicine [25], bioenergy [26], and agriculture [27]. Interestingly, *B. cepacia* is an important conditional pathogen in nosocomial infections because of its wide distribution; while it normally poses no risk to human health, the bacterium tends to attack immunocompromised individuals and often colonizes the lungs of patients with cystic fibrosis or chronic granulomas [25]. *B. cepacia* infection not only causes the progressive deterioration of lung function but can also lead to systemic infections such as necrotizing pneumonia, bacteremia, and sepsis [28]. In recent years, the incidence of clinical isolation of this organism has been on the rise, which raises issues for clinical treatment as it is highly resistant to a variety of antibiotics [25]. The *B. cepacia* complex is also an important group of growth-promoting bacteria that produce extracellular enzymes that can solubilize phosphate in the soil and promote plant growth [29], as well as produce various secondary metabolites such as pyrrolnitrin to inhibit fungal diseases [30].

In this work, we isolated the new strain, *B. cepacia* BRDJ, from the roots of DXWR plants. We performed inoculation experiments to test its growth-promoting potential on different rice cultivars, which was more pronounced in the *japonica* rice than in the *indica* rice, suggesting genetically encoded differences in the rice in response to the same PGPR strain. We sequenced the genome of the strain *B. cepacia* BRDJ and analyzed the mechanisms by which it may promote growth in rice, resulting in the identification of numerous genes. Further analysis focused on the genomic differences between *B. cepacia* phytopathogenic strains and the plant growth–promoting strains. Our study thus identified a beneficial *B. cepacia* strain from the DXWR roots and provides new insights into the potential differentiation principle between the *B. cepacia* phytopathogenic and plant growth–promoting strains.

## 2. Results

### 2.1. Isolation of Rhizobacteria from DXWR

The DXWRs cultivated in the ex situ nursery of Nanchang were propagated from ratoons collected in their original habitat. We collected root samples to isolate the DXWR-associated PGPRs. Accordingly, we washed the roots extensively with sterile water before crushing them in a mortar. The resulting homogenate suspensions were spread on Burk′s nitrogen-free medium to isolate the nitrogen-fixing bacteria [31], leading to the isolation of 101 strains. We selected 10 strains to sequence and compare their 16S rRNA sequences by the BLAST, which revealed three distinct bacterial species. We inoculated the rice plants with each of the three bacteria species and recorded the plant growth. The *Burkholderia* sp. exhibited the strongest plant growth-promoting activity of the three bacterial species tested. We focused on this strain for the further characterization and named this strain BRDJ (*Burkholderia* sp. from Root of DXWR in the Jiangxi province).

### 2.2. Identification of the Strain BRDJ

We determined the sequence of the 16S RNA gene from the BRDJ and used the sequence as a query for the BLAST, which indicated that the strain is closest to *B. cepacia*. We downloaded the 16S RNA sequences of the representative *Burkholderia* spp. from the National Center for Biotechnological Information (NCBI) and aligned these sequences to those from the BRDJ 16S RNA; the alignment showed that the BRDJ is closest to the members of the Bcc (Figure S1A). As the 16S rRNA sequences from the Bcc are relatively close [32], we used the *recA* gene to determine the exact strain [33]. To this end, we constructed a phylogenetic tree for the *recA* sequences from the Bcc and BRDJ, which revealed that the BRDJ is closest to *B. cepacia* ATCC 25416 (Figure S1B). Taken together, these results suggested that the BRDJ is a new *B. cepacia* strain. While *B. cepacia* is a well-known plant growth-promoting and opportunistic pathogen, the plant growth–promoting mechanism in rice has not been thoroughly investigated.

### 2.3. Plant Growth–Promoting Activity of BRDJ

**Figure 1:**
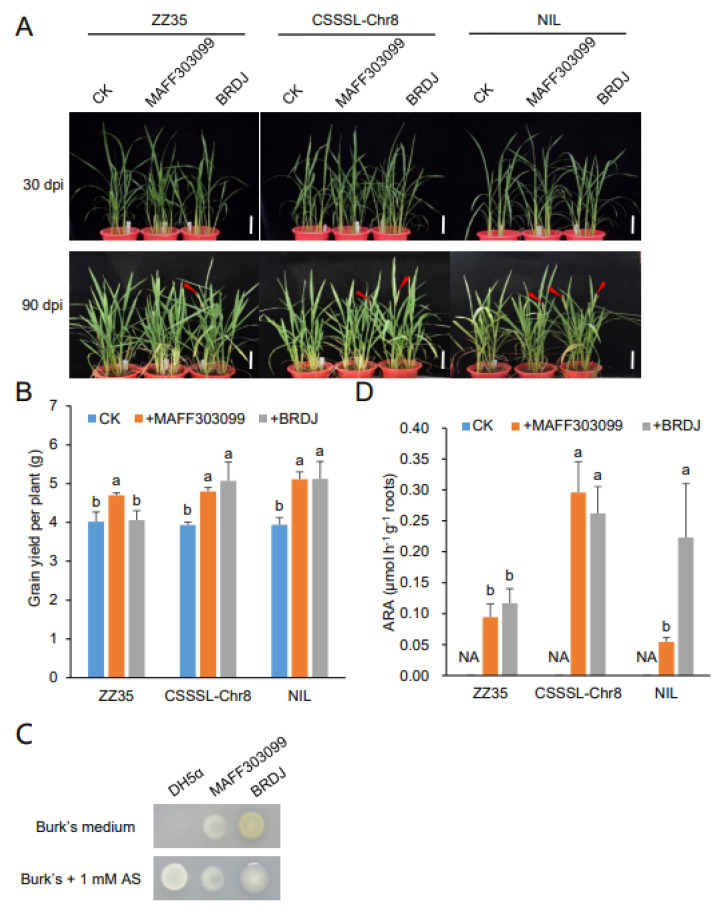
Plant growth promoting activity of BRDJ. (**A**) Representative images of greenhouse experiment. Inflorescence marked by red arrowhead. NIL, near-isogenic line; CSSSL-Chr8, chromosome eight single-segment substitution line. Scale bars, 10 cm. (**B**) Grain yield of harvested rice grown in greenhouse conditions. For each cultivar, nine plants were measured. Values are presented as means ± SD. Different letters indicate significant differences among treatments (Fisher’s Least Significant Difference test, *p*  <  0.05). (**C**) Comparative growth of *Escherichia coli* DH5α, *Mesorhizobium loti* MAFF303099 and *Burkholderia cepacia* BRDJ. 1 mM ammonium sulfate (AS) was added to Burk’s medium as an additional nitrogen source. (**D**) Acetylene-reduction activities (ARA) of excised roots co-cultured with BRDJ or *Mesorhizobium loti* MAFF303099. For each group, three roots were measured. Values are presented as means ± SD. Different letters indicate significant differences among treatments (Fisher’s Least Significant Difference test, *p*  <  0.05). NA, not available.

To investigate the potential growth-promoting effects of this strain, we inoculated plants from the rice cultivar Zhongzao 35 (*O. sativa* L. ssp. *indica*. cv. Zhongzao 35, thereafter ZZ35) with a BRDJ cell culture and compared the growth with rice plants inoculated with the nitrogen-fixing bacterial strain *Mesorhizobium loti* MAFF303099. We noticed that the BRDJ has some ability to promote rice growth, as the inoculated group flowered earlier than the control plants (Figure 1A). We also discovered that a chromosome single-segment substitution line (CSSSL-Chr8), with only one DXWR genomic fragment containing *CHITIN ELICITOR RECEPTOR KINASE 1* (Os*CERK1^DY^*) in an otherwise ZZ35 background [7], grew better than the ZZ35 when inoculated with the BRDJ (Figure 1A,B). BRDJ is able to grow on Burk′s nitrogen-free medium (Figure 1C) and therefore may promote rice growth through nitrogen fixation. We collected the roots from the inoculated rice plants and tested their nitrogen-fixing activity using the acetylene-reduction method [34]. We observed that the plants of the CSSSL-Chr8 line, harboring a small DXWR fragment on chromosome 8 in an otherwise largely ZZ35 background (Figure S2), have higher nitrogen-fixing activity when inoculated with the BRDJ than with the ZZ35 (Figure 1D).

### 2.4. Plant Growth–Promoting Activity of the BRDJ Strain on Different Rice Cultivars

**Figure 2:**
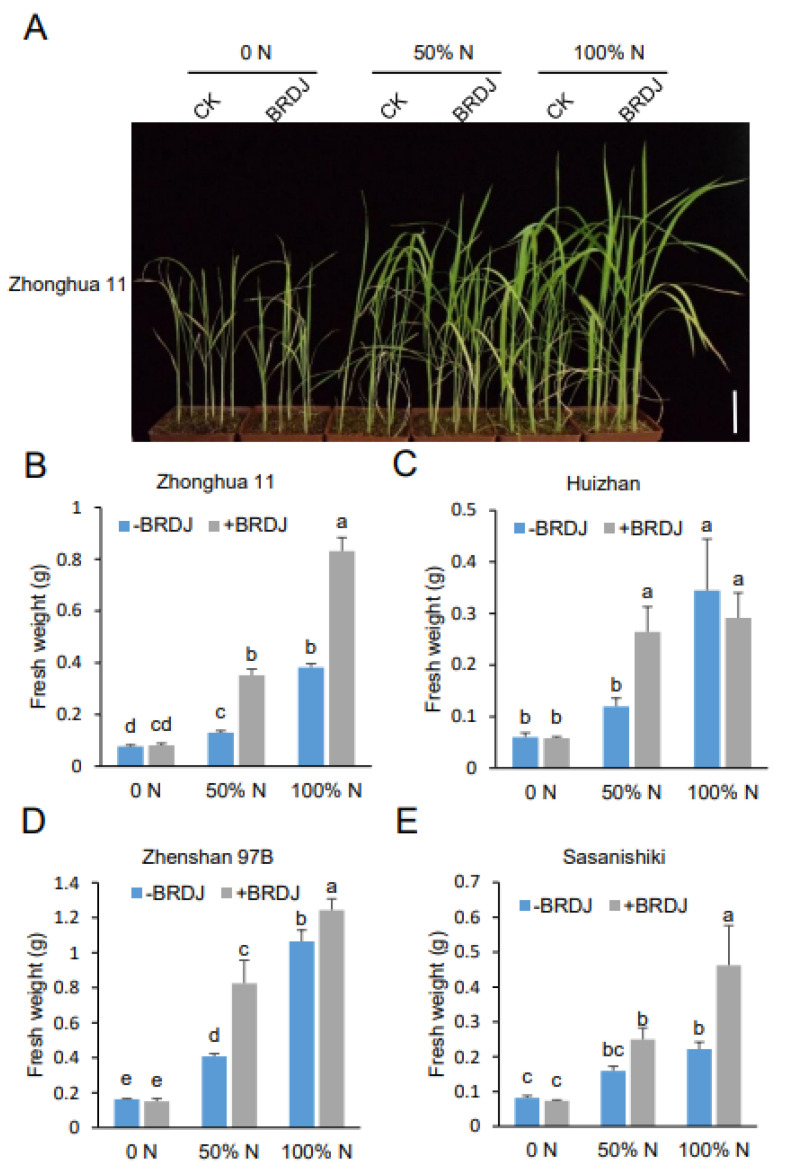
Plant growth-promoting activity of BRDJ on representative rice cultivars. (**A**) Representative images of Zhonghua 11 plants inoculated with BRDJ or mock growing on vermiculite/perlite mix. Scale bar, 5cm. (**B**–**E**) Fresh weight of Zhonghua 11 (**B**), Huizhan (**C**), Zhenshan 97B (**D**), Sasanishiki (**E**) measured at 45 dpi. For each group, 18 plants were measured. Values are presented as means ± SD. Different letters indicate significant differences among treatments (Fisher’s Least Significant Difference test, *p*  <  0.05).

To investigate whether the observed growth promotion extended to other cultivated rice varieties, we tested four additional and representative rice cultivars, including Zhonghua 11 (*japonica*, hereafter referred to as ZH11), Huizhan (*indica*, HZ), Sasanishiki (*japonica*, Sasa), and Zhenshan 97B (*indica*, ZS97B). To this end, we inoculated the plants with the BRDJ and recorded their fresh weight 45 days later. We observed that the fresh weight for two *japonica* varieties were increased under both the 50% and 100% nitrogen Hoagland solution (Figure 2B,E), while the two *indica* varieties showed a growth promotion under the 50% nitrogen conditions but less so under the 100% nitrogen conditions (Figure 2C, D). As DXWR is genetically more closely related to *japonica* rice than to *indica* rice [35], we speculate that this PGPR may be better adapted to the *japonica* varieties. We suggest that the BRDJ promotes growth mainly by improving the nitrogen use efficiency since the BRDJ inoculation did not significantly increase the biomass under the nitrogen-free Hoagland solution treatment (Figure 2B–E).

### 2.5. Identification of Bacterial Plant Growth–Promoting Genes

**Figure 3:**
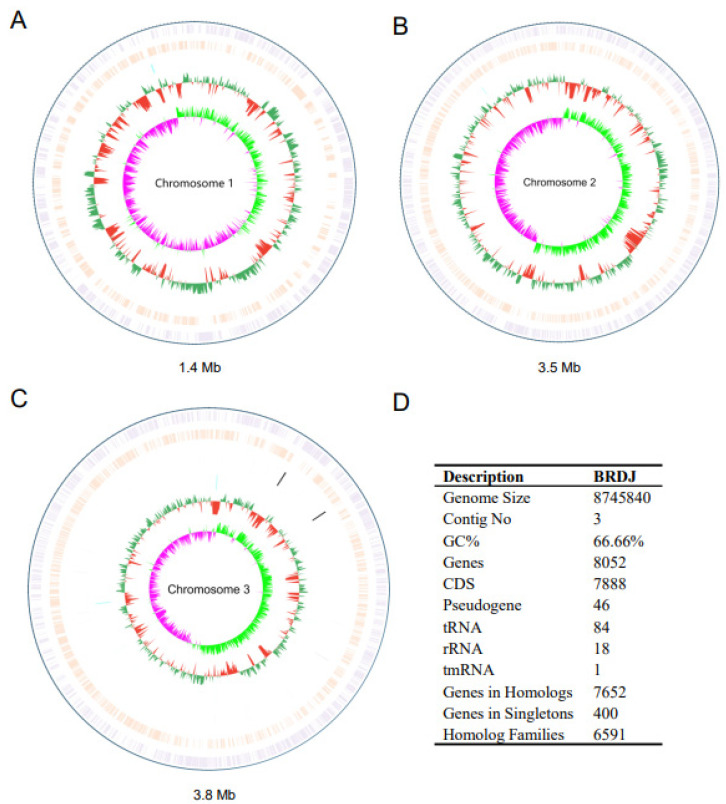
The overview of the BRDJ genome. (**A–C**) The circular map of BRDJ chromosomes. The tracks from outside to center represent the following features: (1) Forward CDS (pale purple); (2) Reverse CDS (apricot); (3) ncRNA (black); (4) %GC plot (green and red correspond to above and below average GC content respectively); (5) GC Skew [(G − C)/(G + C)] (light green and pink correspond to above and below average GC-skew respectively). All features were obtained from available annotations. (**D**) Table of the BRDJ genome properties.

To elucidate the genetic mechanism by which this bacterial strain promotes plant growth, we sequenced the BRDJ genome (Figure 3A–C). The genome of *Burkholderia cepacia* BRDJ consists of three circular chromosomes covering 8,745,840 bp and an average GC content of 66.7%. We predicted 7888 protein-coding genes, 84 transfer RNA (tRNA) genes, and 18 ribosomal RNA (rRNA) genes (Figure 3D). The assembled genome sequence of the strain BRDJ has been deposited in the NCBI under accession number CP095496-CP095498.

We identified many genes in the BRDJ genome that are likely associated with the plant growth–promoting effects (Table 1 and Table S1), such as the genes involved in nitrogen fixation, nutrient uptake, biocontrol, and phytohormone and volatile biosynthesis, or encoding 1-aminocyclopropane-1-carboxylic acid deaminase. We detected the nitrogen fixation–related genes *nifQ*, *nifU*, *nfeD*, *fixA*, *fixB*, *fixJ*, and *fixX*, and the nodulation-related genes *nodJ*, *nodI*, and *nodW* in the BRDJ genome, reflecting its nitrogen fixation function and its ability to grow on a nitrogen-free medium (Figure 1D). It is worth noting that BRDJ may also have a strong iron uptake capacity, since it can synthesize two types of iron chelators, pyochelin (from the *pch* operon genes *pchB*, *pchC*, *pchD*, *pchE*, *pchF*, *pchG*, and *pchR*) and pyoverdin (*pvd* operon genes *pvdA* and *pvdE*). In addition to promoting plant growth by enhancing the nutrient uptake, BRDJ may also secrete a number of growth-promoting substances. Indeed, the BRDJ genome encodes genes related to indole-3-acetic acid (IAA) biosynthesis (*trpA*, *trpB*, *trpC*, *trpD*, *trpE*, *trpG*, and *iaaH*) [36]. In addition to producing phytohormones, BRDJ may also synthesize several growth-promoting substances, including acetoin (*acoA*, *acoB*, and *acoR*), 2,3-butanediol (*butA*, *butB*, *ilvA*, *ilvB*, *ilvC*, *ilvD*, *ilvE*, and *ilvH*), and isoprene (*ispD*, *ispF*, *ispG*, and *ispH*). Moreover, the BRDJ genome harbors biocontrol-related genes including genes encoding chitinase and enzymes involved in phenazine biosynthesis (*phzA*, *phzD*, *phzE*, *phzF*, *phzG*, and *phzI*) and pyrrolnitrin biosynthesis (*prnA*, *prnB*, *prnC*, and *prnD*), which confer plants with greater resistance against diseases [37].

### 2.6. Comparative Genomics Analysis

**Figure 4:**
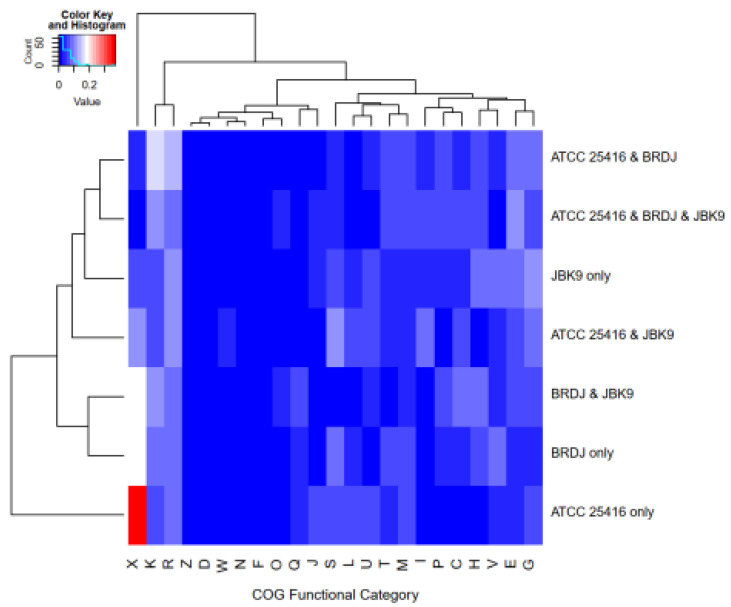
Heatmap based on core and strain-specific clusters of orthologous groups (COGs). The horizontal axis shows the group of shared or unique genes. The vertical axis corresponds to COG functional categories. The data is normalized (number of genes with related functions in the group/total number of genes in the group). COG functional categories: Translation, ribosomal structure and biogenesis (J), Transcription (K), Replication, recombination and repair (L), Cell cycle control, cell division, chromosome partitioning (D), Defense mechanisms (V), Signal transduction mechanisms (T), Cell wall/membrane/envelope biogenesis (M), Cell motility (N), Cytoskeleton (Z), Extracellular structures (W), Intracellular trafficking, secretion, and vesicular transport (U), Posttranslational modification, protein turnover, chaperones (O), Mobilome: prophages, transposons (X), Energy production and conversion (C), Carbohydrate transport and metabolism (G), Amino acid transport and metabolism (E), Nucleotide transport and metabolism (F), Coenzyme transport and metabolism (H), Lipid transport and metabolism (I), Inorganic ion transport and metabolism (P), Secondary metabolites biosynthesis, transport and catabolism (Q), General function prediction only (R), Function unknown (S).

**Figure 5:**
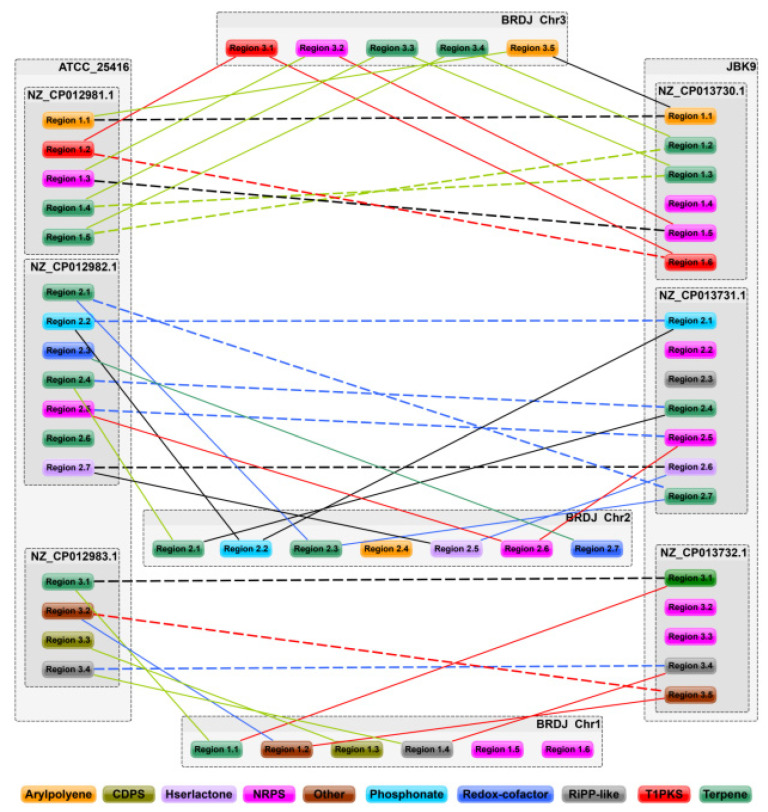
The distribution of secondary metabolite gene clusters in the three genomes. The lines indicate that the two clusters can be matched by the BLAST. The solid lines link the reference genome to the BRDJ, and the dashed lines link the two reference genomes. The different colors correspond to the sequence similarity. Green, 100% coverage; blue, 100% coverage by local alignment; black, 50% to 100%; red, less than 50%. The different types of gene clusters are distinguished by the different colors. CDPS, tRNA-dependent cyclodipeptide synthase; NRPS, non-ribosomal peptide synthetase; RiPP, ribosomally synthesized and post-translationally modified peptide; T1PKS, Type I polyketide synthase.

The *B. cepacia* complex includes both phytopathogenic strains and plant growth–promoting strains. To explore their genetic differences, we compared the BRDJ genome with that from two *B. cepacia* strains, ATCC 25416 and JBK9. The *B. cepacia* strain ATCC 25416, isolated from an onion, can induce bacterial soft rot [38], while the *B. cepacia* JBK9 is a newly identified PGPR strain with antifungal activity [30]. Compared to BRDJ and JBK9, ATCC 25416 has more mobile elements in its unique gene group (Figure 4), which may be responsible for its pathogenicity. Turning to the secondary metabolite gene clusters, we discovered that BRDJ and JBK9 both possess additional unique non-ribosomal peptide synthetase (NRPS) or NRPS-like gene clusters (Figure 5). Several studies showed that non-ribosomal peptides can chelate iron or act as bacterial inhibitors. BRDJ has one more tRNA-dependent cyclodipeptide synthase (CDPS) gene cluster than JBK9 (Figure 5). This gene cluster may confer a stronger resistance to fungal pathogens [39].

### 2.7. Pathogenicity Test in Onions and Nematodes

*B. cepacia* was discovered as a pathogenic bacterium in onions [20]. In order to be applied in agricultural production, the pathogenicity of BRDJ must be evaluated. We chose onion and *Caenorhabditis elegans* as model organisms to compare the pathogenicity of the BRDJ with other bacterial strains. The results showed that the pathogenicity of the BRDJ was significantly reduced compared to the reference strain, ATCC 25416 (Figure S3). After the inoculation of the onions, the ATCC 25416 caused significantly larger maceration areas within 48 h than the BRDJ (Figure S3A). The fast-killing assay also showed that the BRDJ was significantly less pathogenic than the ATCC 25416 in *C. elegans* (Figure S3B), which means that the BRDJ may secrete fewer toxic molecules [40,41]. We next used the PHI (Pathogen Host Interactions, http://www.phi-base.org/; accessed date, 22 August 2019) database for the pathogen gene analysis, and the predicted genes with a sequence identity over 70% are listed in Table S2. Most of these genes are associated with a virulence phenotype. Although the BRDJ showed an attenuated virulence in the onions and *C. elegans*, it may be necessary to edit the pathogenicity-related genes (Table S2) to eliminate the minimal threat it poses before its utilization as a biofertilizer in the field.

## 3. Discussion

In this study, we isolated and characterized the *B. cepacia* strain BRDJ with plant growth–promoting properties from the roots of DXWR. The genome analysis uncovered nodulation- and nitrogen fixation–related genes in the BRDJ strain (Table 1). The atmospheric nitrogen is reduced to ammonia via a biological nitrogen fixation, thus providing nitrogen nutrition to plants. The efficient use of biological nitrogen fixation can reduce the amount of chemical ammonia fertilizer in agricultural production, thus offering a solution to water eutrophication and making agriculture more sustainable [42]. The PGPRs produce nitrogenase that can fix nitrogen from the air for both themselves and the plants, resulting in improved nitrogen nutrition for crops and, thus, higher yields. *Burkholderia* can nodulate with most legumes in the genus *Mimosa* [43]. A phylogenetic analysis reveals that the *Burkholderia*–*Mimosa* symbiosis may be more ancient than we think [44]. The nitrogen-fixing PGPRs such as BRDJ may aid plant growth under low-nitrogen conditions. The BRDJ genome contains multiple copies of the phosphate transporter genes, which contribute to better phosphate nutrition for the wild rice growing in phosphate-poor soils [45]. Although plants take up most of their phosphorus from the soil, much of this phosphate is in an insoluble form that is not readily bioavailable. While chemical fertilizers provide soluble phosphates, they also increase the likelihood that metal cations in the soil will combine with the phosphate to form insoluble salts, thus causing soil consolidation. The use of PGPRs as a biofertilizer will help improve the phosphate nutrient conditions by releasing these insoluble phosphate salts [46]. Iron is the second most abundant metal element in the Earth′s crust and is essential for plant growth, but is not readily bioavailable due to low solubility, especially for the highly oxidized ferric salt. Siderophores can accelerate the dissolution of insoluble ferric salt [47]. The BRDJ genome harbors genes related to the biosynthesis of siderophores, such as pyochelin and pyoverdine (Table S1), suggesting that crops such as rice may absorb these forms of chelated iron via microbial siderophores.

Most PGPRs not only convert insoluble nutrients for plant utilization, but also produce long-acting regulatory substances that improve the nutrient absorption and stress resistance of plants. The BRDJ genome has a gene encoding a 1-aminocyclopropane-1-carboxylic acid (ACC) deaminase on chromosome 2 (Table S1). ACC is the biosynthetic precursor of ethylene, whose levels are determined by those of the ACC. ACC deaminase is an ethylene catabolism enzyme, as it converts the ACC to α-ketobutyric acid and ammonia, thereby indirectly regulating the ethylene biosynthesis. ACC deaminase–producing rhizobacteria can lower the ethylene production by degrading the ACC, thereby increasing the plant drought tolerance and delaying the plant senescence and death [48]. When plants are under constant stress, the ACC secreted by the plant cells is degraded by the ACC deaminase–producing bacteria, thus relieving the inhibitory effects of ethylene on the plant growth. Aside from the ethylene, the BRDJ may directly synthesize the IAA to regulate plant growth, as many PGPRs do.

Many microorganisms can promote rice growth in nature. However, during rice domestication, the genes associated with the pathogen-related PGPRs might be subjected to negative selection, resulting in cultivated rice varieties that display a lower probability of establishing an interaction with pathogenic microbes [49]. The Bcc includes several opportunistic pathogens that are usually resistant to many antibiotics, making them difficult to treat once they have infected the lungs [50]. *B. cepacia* BRDJ, which we isolated from the roots of DXWR, may represent such an example. Compared to the non-pathogenic bacterium *Mesorhizobium loti* strain MAFF303099, the BRDJ conferred a more limited growth-promoting effect to the rice cultivar ZZ35 (Figure 1B), suggesting that this rice cultivar may have lost genes associated with the phytopathogen-related PGPRs. In the inoculation experiments, we determined that two rice lines carrying a DXWR-derived chromosome segment, in an otherwise ZZ35 background fragment, exhibit a stronger growth-promoting effect than in the ZZ35; notably, the plant strains carrying the Os*CERK1^DY^* variant from the DXWR showed stronger acetylene reduction activity after the inoculation with the MAFF303099 (Figure 1D). The Os*CERK1^DY^* allele enhanced the arbuscular mycorrhizal fungi (AMF) symbiosis and increased the resistance against rice blast [7]. Although there is evidence that *Arabidopsis* (*Arabidopsis thaliana*) *CERK1* is involved in bacterial perception through the lysM-motifs [51,52], whether the Os*CERK1^DY^* can enhance the effects of the PGPRs needs to be investigated.

We noticed subtle differences upon the inoculation of *indica* and *japonica* rice with the BRDJ, as this strain did not effectively promote the growth of the *indica* rice (HZ, ZS97B) under the 100% nitrogen conditions. *Indica* and *japonica* rice are associated with distinct microbiomes, suggesting that the domestication of both species was accompanied by a preferential selection for specific microbial communities. The *Indica* roots are more enriched in the microbial taxa associated with the nitrogen cycle than the *japonica* rice roots, resulting in a more active nitrogen transformation environment, and this phenomenon is closely related to the nitrate transporter NRT1.1B [53]. Microbial taxa in the root system of *indica* rice would allow the *indica* rice to have a higher nitrogen fertilizer use efficiency [53]. We also established in our pot experiments that the growth-promoting bacterial strain BRDJ is less effective in the *indica* rice cultivars experiencing the 100% nitrogen conditions, possibly because *indica* rice has a more efficient nitrogen utilization than *japonica* rice, thereby masking the nitrogen supplementation provided by the bacteria. Another possible explanation is that a higher level of cooperation exists between the BRDJ and *japonica* rice, to which the DXWR is more genetically related than *indica* rice [35]. Wild rice is the host of a wide range of endophytic bacteria, as in its seeds. Most of these bacteria showed plant growth–promoting ability, improving the agronomic traits (root length, shoot length, dry mass, and chlorophyll contents) of the rice seedlings.

In addition to the PGPRs, members of the Bcc have been reported to act as animal or plant pathogens. Two representative examples are the sequenced strains of *B. cepacia*, ATCC 25416 and JBK9. ATCC 25416 was isolated from rotting onions and is a pathogenic bacterium that can cause damage to onions or human epithelial cells [38,54]. Compared to the ATCC 25416, the BRDJ showed a significantly attenuated pathogenicity in onions (Figure S3A). A majority of the predicted pathogenicity-related genes are associated with a virulence reduction (Table S2). JBK9 was obtained from the rhizosphere of cultivated crops and exhibits a biocontrol and plant growth–promoting capacity. Indeed, a JBK9 culture is effective in controlling against the *Phytophthora* blight in red peppers (*Capsicum annuum*) by producing pyrrolnitrin. A comparative genomic analysis showed that the ATCC 25416 genome possesses more unique mobile elements than the BRDJ or JBK9. The mobilome consists of mobile genetic elements such as transposons, bacteriophages, and self-splicing introns [55]. We propose that these mobile genetic elements were the intrinsic driver of the *B. cepacia* evolution toward phytopathogenicity. These active elements may disrupt some crucial genes in the ATCC 25416 genome, increasing the need to acquire nutrients from the host to such an extent that it kills the host, rather than establishing a beneficial relationship. This hypothesis is supported by a comparison of ATCC 25416–specific clusters of orthologous groups (COGs) with the BRDJ and JBK9. The BRDJ-specific and JBK9-specific COGs contain a larger proportion of genes associated with the transport and metabolism of carbohydrates, amino acids, and coenzymes (Figure 4). This inference may also apply to other bacteria with diverged lifestyles such as *Salmonella*, *Aeromonas*, and *Paracoccus* [56,57,58,59].

## 4. Materials and Methods

### 4.1. Plant Materials and Growth Conditions

The rice seeds (*O. sativa* L. cv. Zhongzao 35, Zhonghua 11, Huizhan, Sasanishiki, and Zhenshan 97B) were surface-sterilized in 2.5% sodium hypochlorite for 30 min, and then washed three times with sterilized water. After germination in sterilized water for 3 d, the plants were transferred to pots with a corresponding substrate. For Figure 1, the rice plants were cultivated in sterilized field soil. For Figure 2, the rice plants were cultivated in a 2:1 mixture of vermiculite and perlite and grown in a greenhouse of 16 h light/8 h dark cycles at 28 °C. The Hoagland solution with a corresponding concentration of nitrogen was added to the pots twice a week.

### 4.2. Isolation of Rhizobacterium Strains

The samples of Dongxiang wild rice roots were collected from the Dongxiang wild rice ex situ nursery in Nanchang, Jiangxi Province (115°56′ E, 28°34′ N). Healthy plants were randomly selected for the sampling, and the plants and the inter-root soil attached to their roots were taken back to the laboratory in sterile bags. The roots were gently shaken in sterile water to remove the larger soil particles and plant residues adhering to the roots. The roots were rinsed three times with sterile water and ground thoroughly using a sterilized mortar. The homogenate was diluted in a gradient, after which 0.1 mL of the dilution was spread onto plates containing Burk′s nitrogen-free solid medium (composition/L: sucrose, 20.0 g; K_2_HPO_4_, 0.64 g; KH_2_PO_4_, 0.16 g; MgSO_4_·7H_2_O, 0.20 g; NaCl, 0.20 g; CaSO_4_·2H2O, 0.05 g; Na_2_MoO_4_·2H_2_O, 0.0025 g; FeSO_4_·7H_2_O, 0.015 g; and agar, 15 g) and incubated at 28 °C for 2 weeks. The plates were then examined to identify the different types of colonies.

### 4.3. Inoculation Experiments in Rice

The rice seeds were surface-sterilized as follows: the seeds were rinsed three times in sterile distilled water, followed by rinsing in 70% (*v/v*) ethanol for 5 min. The seeds were then rinsed in sterile distilled water and then washed in 2.5% sodium hypochlorite (*w/v*) for 30 min. After draining the ethanol, the seeds were rinsed three times with sterile water. Finally, the seeds were placed on wet sterile filter paper for germination. The germinated seedlings were transplanted to a sterile vermiculite and perlite mixture (1:1, *w/w*) for 25 days. The growth chamber was set to 28 °C with a photoperiod of 14 h light/10 h dark. The subsequent treatments were carried out using the BRDJ. The BRDJ cultures were inoculated in a YEB medium and incubated at 28 °C with shaking at 180 rpm. The bacterial cultures were resuspended in sterile distilled water to a final OD_600_ of 0.8 and used to inoculate the rice plants.

### 4.4. Nitrogenase Activity Assay

The nitrogenase activity was measured by the acetylene-reduction method. The rice roots co-cultured with the BRDJ were placed in glass vials sealed with rubber plugs. Each bottle contained five root samples, with three biological replicates per variety. Two milliliters of acetylene were injected into each bottle. All the bottles were then incubated at 28 °C for two days. The ethylene production was measured using a GC-4000A gas chromatograph (East & West Analytical Instruments, Beijing, China). For the comparative growth experiment, three strains containing *Escherichia coli* DH5α, *Mesorhizobium loti* MAFF303099 and *Burkholderia cepacia* BRDJ were precultured in a YEB medium, centrifuged at 4000× *g* for 5 min and resuspended in sterilized water until the OD_600_ reached 0.7, and 3 µL of the corresponding suspensions were dropped on Burk′s nitrogen-free solid medium with or without 1mM ammonium sulfate.

### 4.5. Whole-Genome Sequencing

The genomic DNA was extracted with the SDS method [60]. The quality of the extracted DNA was determined by agarose gel electrophoresis and quantified on a Qubit^®^ 2.0 Fluorometer (Thermo Scientific, Waltham, MA, USA). The sequencing libraries for the single-molecule real-time (SMRT) sequencing were constructed with an insert size of 10 kb using the SMRTbell^TM^ Template Kit, version 1.0 (Pacific Biosciences, Menlo Park, CA, USA), according to the manufacturer’s instructions. Briefly, the genomic DNA was fragmented and concentrated, followed by an end-repair and blunt ligation. The resulting SMRTbell Templates were purified with 0.45X AMPure PB Beads (Agencourt, Beverly, MA, USA), size-selected using the BluePippin System (Sage Science, Beverly, MA, USA). Finally, the library concentration was assessed on a Qubit^®^ 2.0 Fluorometer (Thermo Scientific, Waltham, MA, USA) and the insert fragment determined on an Agilent 2100 Bioanalyzer (Agilent Technologies, Palo Alto, CA, USA).

For sequencing on the Illumina NovaSeq platform (Illumina, Foster City, CA, USA), 1 μg of the genomic DNA per sample was used as the input material for the DNA sample preparations. The sequencing libraries were generated using the NEBNext^®^ Ultra™ DNA Library Prep Kit for Illumina (NEB, Ipswich, MA, USA), following the manufacturer’s recommendations. Index codes were added to assign sequences to each sample. Briefly, the DNA samples were fragmented by sonication to a size of 350 bp, then the DNA fragments were end-polished, A-tailed, and ligated with the full-length adaptor for the Illumina sequencing with further PCR amplification. The PCR products were then purified (AMPure XP system) and the libraries were analyzed for the size distribution on an Agilent2100 Bioanalyzer and quantified using quantitative PCR.

The whole genome of the BRDJ was sequenced using the PacBio Sequel platform and Illumina NovaSeq as 150-bp paired-end reads at Beijing Novogene Bioinformatics Technology Co., Ltd.

### 4.6. Genome Assembly

The low-quality reads (<500 bp) were filtered to obtain clean data. Using the automatic error correction function of the SMRT portal, the long reads were selected (more than 6000 bp) as the seed sequence, and the shorter reads were aligned to the seed sequence by BLASR to improve the accuracy of the seed sequence and generate a preliminary assembly. In the variant Caller module of the SMRT Link software, the arrow algorithm was used to correct and count the variant sites in the preliminary assembly. The corrected assembly, which was used as the reference sequence, was used as the reference to align the Illumina reads by BWA [61]. The Illumina reads were filtered with a base minimum mass value of 20, a minimum read depth of 4, and a maximum read depth of 1000.

### 4.7. Genome Component Prediction

The following genome components were predicted: coding genes, repetitive sequences, noncoding RNA, genomic islands, transposons, prophages, and clustered regularly interspaced short palindromic repeat (CRISPR) sequences. The bacterial coding sequences were retrieved with the GeneMarkS program [62]. The interspersed repetitive sequences were predicted using RepeatMasker (http://www.repeatmasker.org/, accessed on 21 August 2019). The tandem repeats were analyzed with the Tandem repeats finder (https://tandem.bu.edu/trf/trf.html, accessed on 21 August 2019). The transfer RNA (tRNA) genes were predicted by the tRNAscan-SE [63]. The ribosomal RNA (rRNA) genes were identified with the rRNAmmer [64]. The small nuclear RNAs (snRNAs) were predicted by the BLAST against the Rfam database [65]. The IslandPath-DIMOB program was used to predict the genomic islands [66], while transposon PSI was used to predict the transposons based on homology by the BLAST. The PHAST was used for the prophage predictions (http://phast.wishartlab.com/, accessed on 22 August 2019).

### 4.8. Gene Function Prediction

The gene functions were predicted using relevant databases: the GO (Gene Ontology, http://geneontology.org/, accessed on 22 August 2019), KEGG (Kyoto Encyclopedia of Genes and Genomes, https://www.genome.jp/kegg/, accessed on 22 August 2019), COG (Clusters of Orthologous Groups, https://www.ncbi.nlm.nih.gov/research/cog/, accessed on 22 August 2019), NR (Non-Redundant Protein Database, https://www.ncbi.nlm.nih.gov/refseq/about/nonredundantproteins/, accessed on 22 August 2019), TCDB (Transporter Classification Database, https://tcdb.org/, accessed on 22 August 2019), PHI (Pathogen Host Interactions, http://www.phi-base.org/, accessed on 22 August 2019) and Swiss-Prot (https://www.sib.swiss/swiss-prot, accessed on 22 August 2019). A whole-genome BLAST search (E-value below 1e–5, with a minimal alignment of at least 40% over the length of the query) was performed against the above six databases. The secretory proteins were predicted by the Signal Peptide database (http://www.signalpeptide.de/, accessed on 22 August 2019), and the prediction of the Type I-VII proteins secreted by the pathogenic bacteria were based on the EffectiveT3 software (https://effectors.csb.univie.ac.at/method/effectivet3, accessed on 22 August 2019). The secondary metabolism gene clusters were analyzed with the antiSMASH algorithm [67]. The carbohydrate-active enzymes were predicted against the Carbohydrate-Active enZYmes Database [68].

### 4.9. Comparative Genomics Analysis

The comparative genomic analysis included the genome synteny, core and strain-specific genes, phylogenetic analysis of gene families, SNP (single nucleotide polymorphism), InDel (insertion and deletion) and SV (structural variation) annotations, and genome visualization. The genomic alignments between the sample genome and reference genomes (or more than two sample genomes) were performed using the MUMmer [69] and LASTZ (http://www.bx.psu.edu/~rsharris/lastz/, accessed on 21 March 2022) tools. The genomic synteny was assessed based on the alignment results. The core genes and specific genes were analyzed by the CD-HIT rapid clustering of similar proteins software [70], with a threshold of a 50% pairwise identity and 0.7 length difference cutoff in the amino acid sequences. Venn diagrams were then drawn to show the relationships across the samples. The polymorphisms (SNPs, InDels, and SVs) were detected from the genome alignments with the MUMmer and LASTZ. The circular plots were created in the Circos [71]. The phylogenetic tree was constructed with the Mega X [72].

### 4.10. Pathogenicity Test on Onion and Nematode

Healthy yellow globe onions (*Allium cepa* L.) were washed several times with sterile water and surface-sterilized with 75% ethanol, then cut by a sterile knife. A deep hole of about 5 mm was made with a 10 μL pipette tip on each slice, and a 10 μL culture of the corresponding strain was inoculated. The inoculated slices were placed in a sterile box incubated at 28 °C for 48 h.

*Caenorhabditis elegans* strain N2 nematodes were fed on an NG medium (0.3% NaCl, 0.25% peptone, 2% agar, 5 μg/mL cholesterol, 1 mM CaCl_2_, 1 mM MgSO_4_, 25 mM K_2_HPO_4_, pH 6.0) using *E. coli* OP50 as the food source at 22 °C. The overnight bacterial cultures were resuspended in sterile distilled water to a final OD_600_ of 1. A 10 μL bacterial suspension was spread on a PGS medium (1% peptone, 1% NaCl, 1% glucose, 0.15 M sorbitol, 1.7% Agar) and incubated at 28 °C for 24 h to form a bacterial lawn. Then, 40–60 synchronized L4 larvae were prepared as described in [40,41] and transferred to the PGS medium. The nematode numbers were scored every 12 h or 24 h. Each experiment was performed with three to four replicates.

## Figures and Tables

**Table 1:** Genes linked to nodulation and nitrogen fixation in BRDJ genome.

PGP Activities	Gene Name	Gene Annotation	Chromosomal Location
**Nodulation**			
	*nodW*	two component transcriptional regulator, Nodulation protein W	Chr1:564266-564919−
	*nodT*	NodT family protein	Chr1:957928-959382+
	*nodS*	Nodulation protein S	Chr3:1174329-1175027−
	*nodJ*	Nodulation protein J	Chr3:1903018-1903851−
	*nodI*	Nod factor export ATP-binding protein I	Chr3:1903858-1904772−
	*nfeD*	Nodulation efficiency protein NfeD	Chr3:2432325-2432759−
**Nitrogen Fixation**			
	*fixX*	Ferredoxin-like protein FixX	Chr1:906003-907661−
	*nifQ*	nitrogen fixation protein NifQ	Chr1:1372743-1373342−
	*fixB, etfA*	electron transfer flavoprotein alpha	Chr2:2094197-2095141−
	*fixA, etfB*	subunit electron transfer flavoprotein beta subunit	Chr2:2095158-2095913−
	*rnfH*	Protein RnfH	Chr3:2429820-2430143−
	*iscU, nifU*	nitrogen fixation protein NifU and related proteins	Chr3:2612558-2612965−
	*iscS, NFS1*	cysteine desulfurase, Nitrogen Fixation 1	Chr3:2613034-2614224−
	*fixJ*	Transcriptional regulatory protein FixJ	Chr3:2631016-2631654+
	*rnfB*	electron transport complex protein RnfB	Chr3:2897345-2898232+
	*nifH*	4Fe-4S iron sulfur cluster binding proteins, NifH/frxC family	Chr3:2990786-2991844−

## Data Availability

The assembled genome sequence of the strain BRDJ has been deposited in the NCBI under accession numbers CP095496, CP095497, and CP095498.

## Supplementary Materials

Supplementary materials can be found at https://www.mdpi.com/article/10.3390/ijms231810769/s1.

## Abbreviations

ACC	1-aminocyclopropane-1-carboxylic acid
AS	Ammonium sulfate
AMF	Arbuscular mycorrhizal fungi
Bcc	*Burkholderia cepacia* complex
CDPS	tRNA-dependent cyclodipeptide synthases
COGs	Clusters of Orthologous Groups
DXWR	Dongxiang wild rice
IAA	Indole-3-acetic acid
NRPS	Non-ribosomal peptide synthetase
RiPP	Ribosomally synthesized and post-translationally modified peptide
T1PKS	Type I polyketide synthase
YEB	Yeast Extract Beef
