# Supplementary material for: A Wild Rice Rhizobacterium Burkholderia cepacia BRDJ Enhances Nitrogen Use Efficiency in Rice
Source: Int J Mol Sci. 2022 Sep 15;23(18):10769. doi: 10.3390/ijms231810769 (PMC9503923; doi:10.3390/ijms231810769)
Supplement: Supplementary file 1 [file ijms-23-10769-s001.zip › ijms-1840260-supplementary.pdf]

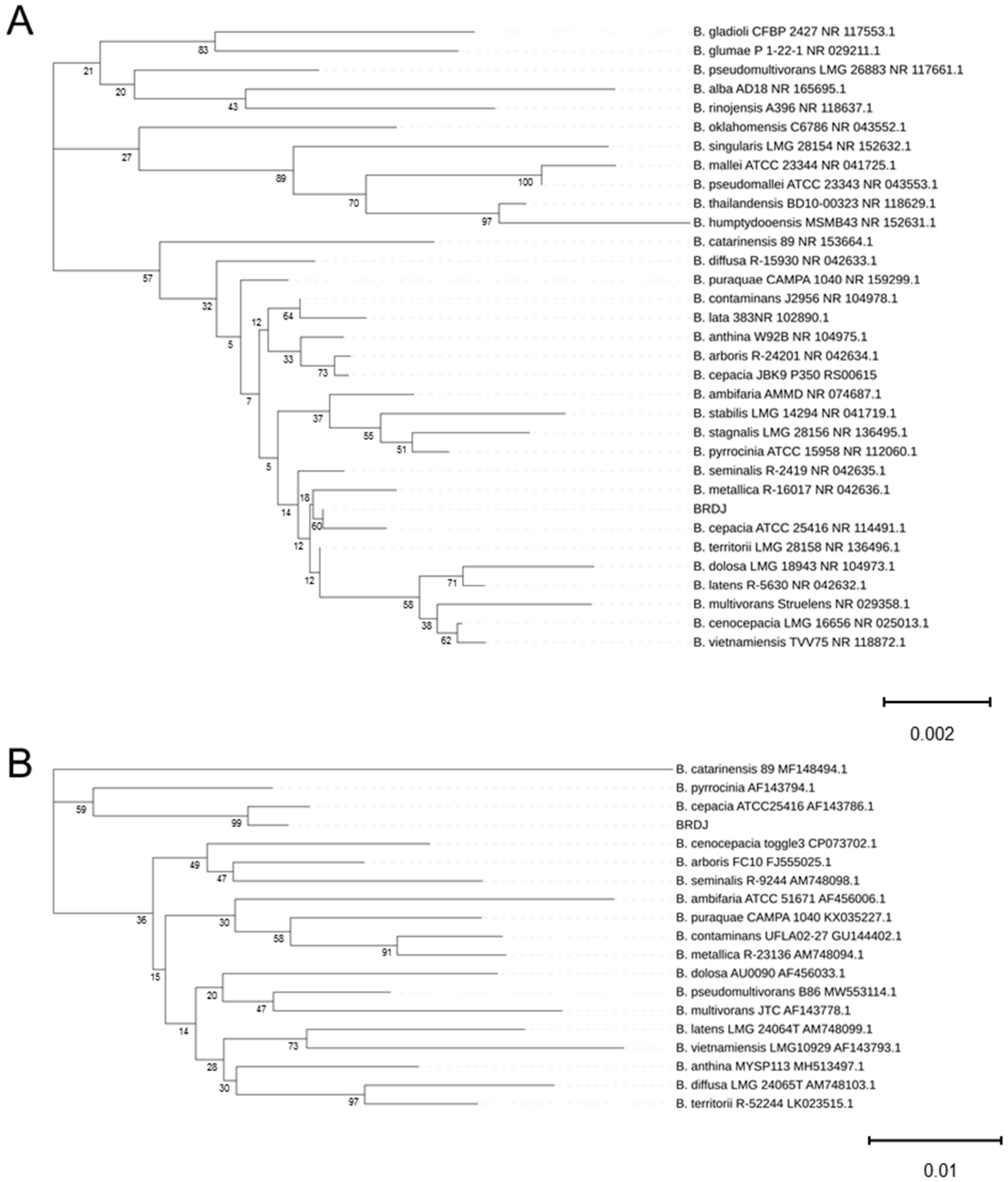

Figure S1. Phylogenetic analysis of the strain BRDJ. (A) Phylogenetic tree based on 16S rRNA using the neighbor-joining method to compare BRDJ to other strains inside the *Burkholderia*. The scale bar indicates the expected changes per site substitution per nucleotide. The bootstrap values (%) based on 1000 bootstrap replicates are marked on each node. (B) Phylogenetic tree based on *recA*. The scale bar indicates the expected changes per site substitution per nucleotide. The bootstrap values (%) based on 1000 bootstrap replicates are marked on each node.

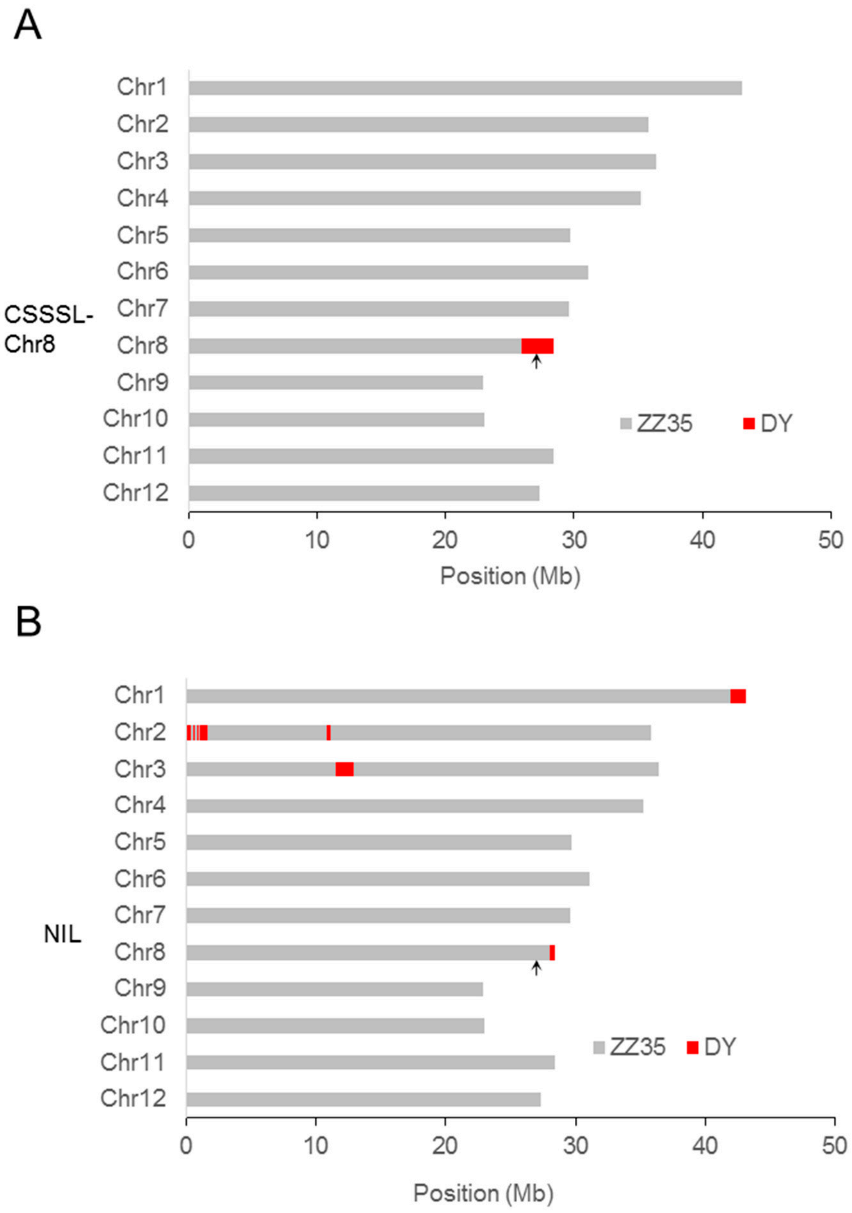

Figure S2. Diagram of chromosome composition for CSSSL-Chr8 (A) and NIL (B). NIL, near-isogenic line; CSSSL-Chr8, chromosome 8 single-segment substitution line. The arrows indicate the position of Os*CERK1*^{DY} and Os*CERK1*^{ZZ35}, respectively.

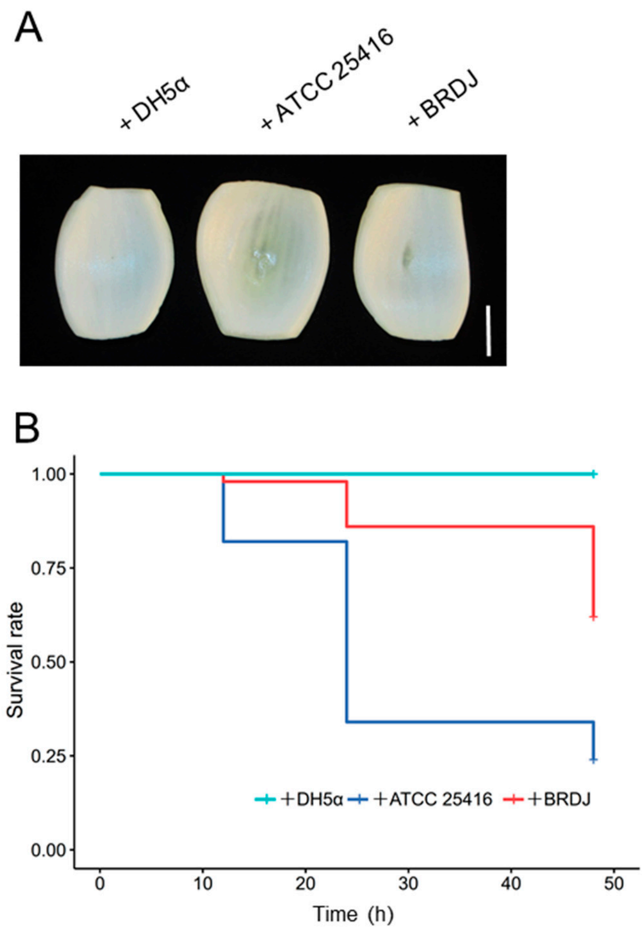

Figure S3 Pathogenicity test on onion and worm.

(A) Tissue maceration symptom of onion slices after 48 h incubation at 28℃. Onion slices were wounded by pipette tip, 10μL culture of the corresponding strain was inoculated. The experiments were performed three times. One of the representative results is shown. Scale bar, 2 cm. (B) Survival curve of *Caenorhabditis elegans* in a fast-killing assay. The experiment were repeated three times. One of the representative results is shown. The curves were plotted using the Kaplan–Meier method. The log-rank test showed a significant difference ($P < 0.0001$) between ATCC 25416 and BRDJ treatment.

Table S1. Genes linked to plant growth promoting and biocontrol activities in the strain BRDJ genome.

PGP Activities	Gene name	Gene Annotation	Chromosome Location
**ACC Deaminase**			
	acdS	1-aminocyclopropane-1-carboxylate deaminase	Chr2:409477-410493 -
**Siderophores**			
Pyochelin	*pchG*	pyochelin biosynthetic protein PchG	Chr2:2791298-2792350 -
	pchF	pyochelin synthetase	Chr2:2792347-2797821 -
	pchE	dihydroaeruginoic acid synthetase	Chr2:2797818-2802140 -

Table S1 Continued

PGP Activities	Gene name	Gene Annotation	Chromosome Location

	pchR	AraC family transcriptional regulator, transcriptional activator of the genes for pyochelin and ferripyochelin receptors	Chr2:2802351-2803250 -
	pchD	pyochelin biosynthesis protein PchD	Chr2:2803531-2805162 +
	pchC	pyochelin biosynthetic protein PchC	Chr2:2805159-2805932 +
	pchB	isochorismate pyruvate lyase	Chr2:2805929-2806234 +
Pyoverdin	*pvdE*	putative pyoverdin transport system ATP-binding/permease protein	Chr3:1962159-1963904 -
	pvdA, *SIDA*	L-ornithine N5-monooxygenase	
**Phosphate Metabolism**			
	pstS	phosphate transport system substrate-binding protein	Chr3:1457432-1458466 +
	pstC	phosphate transport system permease protein	Chr3:1458561-1459547 +
	pstA	phosphate transport system permease protein	Chr3:1459544-1460437 +
	pstB	phosphate transport system ATP-binding protein	Chr3:1460459-1461301 +
	phoU	phosphate transport system protein	Chr3:1461320-1462024 +
	phoB	two-component system, OmpR family, phosphate regulon response regulator PhoB	Chr3:1462057-1462758 +
	phoR	two-component system, OmpR family, phosphate regulon sensor histidine kinase PhoR	Chr3:1462816-1464135 +
	pstS	phosphate transport system substrate-binding protein	Chr3:1747532-1748254 +
	phoH, *phoL*	phosphate starvation-inducible protein PhoH and related proteins	Chr3:3272707-3273753 +
**Plant Hormones**			
	iaaH	indoleacetamide hydrolase	Chr1:664183-665646 -
	trpB	tryptophan synthase beta chain	Chr2:1160344-1161537 +
	trpA	tryptophan synthase alpha chain	Chr2:1162439-1163254 +

Table S1 Continued

PGP Activities	Gene name	Gene Annotation	Chromosome Location
	trpC	indole-3-glycerol phosphate synthase	Chr3:576638-577423 -
	trpD	anthranilate phosphoribosyltransferase	Chr3:577453-578484 -
	trpG	anthranilate synthase component II	Chr3:578498-579088 -
	trpE	anthranilate synthase component I	Chr3:579102-580595 -
**Volatiles**			
Acetoin	*acoR*	sigma-54 dependent transcriptional regulator, acetoin dehydrogenase operon transcriptional activator AcoR	Chr2:2759917-2761821 +
	acoB	acetoin:2,6-dichlorophenolindophenol oxidoreductase subunit beta	Chr3:2260355-2261359 -
	acoA	acetoin:2,6-dichlorophenolindophenol oxidoreductase subunit alpha	Chr3:2261414-2262397 -
2,3-Butanediol	*BDH*, *butB*	(R,R)-butanediol dehydrogenase / meso-butanediol dehydrogenase / diacetyl reductase	Chr2:127771-128892 -

	butA, *budC*	meso-butanediol dehydrogenase / (S,S)-butanediol dehydrogenase / diacetyl reductase	Chr3:2262475-2263251 -
	ilvC	ketol-acid reductoisomerase	Chr3:2824437-2825453 -
	ilvH, *ilvN*	acetolactate synthase I/III small subunit	Chr3:2825558-2826049 -
	ilvB, *ilvG*, *ilvI*	acetolactate synthase I/II/III large subunit	Chr3:2826161-2827843 -
	ilvD	dihydroxy-acid dehydratase	Chr3:3020573-3022246 +
	ilvE	branched-chain amino acid aminotransferase	Chr3:3215940-3216857 +
	ilvA, *tdcB*	threonine dehydratase	Chr3:3322555-3324078 +
Isoprene	*ispH*, *lytB*	4-hydroxy-3-methylbut-2-en-1-yl diphosphate reductase	Chr2:3423723-3424478 +
	gcpE, *ispG*	(E)-4-hydroxy-3-methylbut-2-enyl-diphosphate synthase	Chr3:2231962-2233212 -
	ispF	2-C-methyl-D-erythritol 2,4-cyclodiphosphate synthase	Chr3:2374823-2375308 -
	ispD	2-C-methyl-D-erythritol 4-phosphate cytidylyltransferase	Chr3:2375337-2375984 -

Table S1 Continued

PGP Activities	Gene name	Gene Annotation	Chromosome Location
**Biocontrol**			
Chitinase		chitinase	Chr3:2009034-2010389 -
Phenazine	*phzG*	dihydrophenazinedicarboxylate synthase	Chr1:891157-891795 -
	phzF	trans-2,3-dihydro-3-hydroxyanthranilate isomerase	Chr1:891833-893041 -
	phzE	2-amino-4-deoxychorismate synthase	Chr1:893086-895017 -
	phzD	trans-2,3-dihydro-3-hydroxyanthranilic acid synthase	Chr1:895014-895637 -
	phzA_B	phenazine biosynthesis protein	Chr1:895661-896146 -
	rhlI, *phzI*, *solI*, *cepI*, *tofI*	acyl homoserine lactone synthase	Chr2:2466342-2466875 +
Pyrrolnitrin	*prnA, rebH, ktzQ*	tryptophan 7-halogenase	Chr1:235213-236829 +
	prnB	monodechloroaminopyrrolnitrin synthase	Chr1:236829-237914 +
	ctcP, *cts4*, *prnC*	tetracycline 7-halogenase / FADH2 O2-dependent halogenase	Chr1:237973-239673 +
	prnD	aminopyrrolnitrin oxygenase	Chr1:239698-240810 +

Table S2. Animals and plants pathogenicity-related genes in the strain BRDJ genome.

Host species	Gene Name	Identity	Gene Function	Mutant Phenotype	Disease Name	Chromosome Location
**Plants**						
Allium cepa	PidS	84.6	Sensor histidine kinase / two-component regulatory system	reduced virulence	Panicle blight	Chr3:110284-111750-
	PidR	98.7	Response regulator/ two-component regulatory	reduced virulence	Panicle blight	Chr3:111887-112579-
	qsmR	82.3	IclR-type transcriptional regulator	reduced virulence	Bacterial panicle blight of rice	Chr3:1344746-1345570-

	flhD	90	Flagellar transcriptional regulator	reduced virulence	Bacterial panicle blight of rice	Chr3:225050-225352+
Medicago sativa	BceR	83.3	response regulator	unaffected pathogenicity	Nosocomial infections	Chr1:1017605-1018252+
	BceR	100	response regulator	unaffected pathogenicity	Nosocomial infections	Chr2:1839081-1839728-

Table S2 Continued

Host species	Gene Name	Identity	Gene Function	Mutant Phenotype	Disease Name	Chromosome Location
Oryza sativa	tofR	77.8	Produce toxoflavin	reduced virulence	Panicle blight	Chr2:2464820-2465539-
	GyrA	93.8	DNA gyrase	chemistry target: resistance to chemical	Bacterial grain rot	Chr3:1176426-1178981+
	aroA	88.7	3-phosphoshikimate 1-carboxyvinyltransferase	reduced virulence	Panicle blight	Chr3:1183145-1184449+
	raxP	70.1	Sulfate adenylyltransferase required for Ax21 activity	increased virulence	Bacterial blight	Chr3:2728642-2729526+
	aroB	86.1	3-dehydroquinate synthase	reduced virulence	Panicle blight	Chr3:406616-407695+
Pyrus communis	tssC-1	76.8	T6SS-1 component	unaffected pathogenicity	Fire blight	Chr2:1914899-1916407+
Raphanus sativus	XC_2203	72.3	Nucleotide diphosphate kinase	reduced virulence	Black rot disease	Chr3:2235768-2236193-
Solanum lycopersicum	fabG1	80.2	3-ketoacyl-acyl carrier protein reductase	lethal	Bacterial wilt	Chr3:1259785-1260534+
**Animals**						
Anas platyrhynchos	cheA	74.4	Chemotaxis protein	unaffected pathogenicity	Meningitis	Chr3:228533-230806+
	cheW	71.8	Chemotaxis protein	unaffected pathogenicity	Meningitis	Chr3:230858-231373+
Caenorhabditis elegans	oqxB	71	Multidrug efflux pump	reduced virulence	Pneumonia	Chr2:2491195-2494407-
	cstA	70.6	Peptide-utilizing carbon starvation protein	reduced virulence	Salmonellosis	Chr2:3432824-3434902-
	paaE	98.1	phenylacetic acid catabolism	reduced virulence	Cystic fibrosis-like conditions	Chr3:338946-340034-
	paaD	95.2	phenylacetic acid catabolism	reduced virulence	Cystic fibrosis-like conditions	Chr3:340040-340600-
	paaC	94	phenylacetic acid catabolism	reduced virulence	Cystic fibrosis-like conditions	Chr3:340624-341427-
	paaB	98.9	phenylacetic acid catabolism	reduced virulence	Cystic fibrosis-like conditions	Chr3:341438-341722-
	paaA	98.2	phenylacetic acid catabolism	reduced virulence	Cystic fibrosis-like conditions	Chr3:341760-342758-
Galleria mellonella	atsR	90.4	global virulence regulator	increased virulence (hypervirulence)	Cystic fibrosis-like conditions	Chr2:394965-396725-

	BceD	98	Protein tyrosine phosphatases	reduced virulence	Cystic fibrosis-like conditions	Chr2:1011100-1011543+
	BCAM1331	88.2	Tyrosine kinases	reduced virulence	Cystic fibrosis-like conditions	Chr2:1629150-1631366+

Table S2 Continued

**Host species**	**Gene Name**	**Identity**	**Gene Function**	**Mutant Phenotype**	**Disease Name**	**Chromosome Location**
	flmQ	87.9	flagellin glycosyltransferase	reduced virulence	Cystic fibrosis-like conditions	Chr3:208838-211177-
	BCAL2200	95	Protein tyrosine phosphatases	reduced virulence	Cystic fibrosis-like conditions	Chr3:2614977-2615459-
	ntrC	93.1	activator protein	unaffected pathogenicity	Cystic fibrosis-like conditions	Chr3:2682121-2683638-
	BCAL0960	94	O-oligosaccharyltransferase	reduced virulence	Cystic fibrosis	Chr3:3225197-3226951-
Homo sapiens	BceF	96.8	Tyrosine Kinase	reduced virulence	Cystic fibrosis	Chr2:1012766-1014991+
Locusta migratoria	MakatG1	70.5	Catalase-peroxidase	unaffected pathogenicity	No data found	Chr3:799462-801648+
Mus musculus	pchB	70.3	Isochorismate pyruvate lyase	unaffected pathogenicity	Infections	Chr2:2805929-2806234+
	VK055_1952	73.1	Betaine aldehyde dehydrogenase	unaffected pathogenicity	Pneumonia	Chr2:2916874-2918343+
	wcbS	92.5	Capsule	reduced virulence	Melioidosis	Chr3:893383-894300+
	wcbT	89.3	Capsule	reduced virulence	Melioidosis	Chr3:894365-895693+
	lsfA	80.2	1-Cys peroxiredoxin with thioldependent peroxidase activity	reduced virulence	Opportunistic infections	Chr3:920428-921066+
	Hfq	88.3	RNA Chaperone	reduced virulence	Pertussis	Chr3:2226753-2227004-
	clpP	74	ATP-dependent Clp protease proteolytic subunit	reduced virulence	Nosocomial infections	Chr3:2355346-2355972-
	BPSL2105	92	Putative phage-shock protein A (PspA) homologue	reduced virulence	Melioidosis	Chr3:2385713-2386393+
